# Tracking the Replication-Competent Zika Virus with Tetracysteine-Tagged Capsid Protein in Living Cells

**DOI:** 10.1128/jvi.01846-21

**Published:** 2022-03-14

**Authors:** Shimin Li, Dianbing Wang, Abbas Ghulam, Xia Li, Min Li, Qin Li, Yingxin Ma, Lei Wang, Hangshen Wu, Zongqiang Cui, Xian-En Zhang

**Affiliations:** a National Laboratory of Biomacromolecules, Institute of Biophysics, Chinese Academy of Sciences, Beijing, China; b Faculty of Synthetic Biology, Shenzhen Institute of Advanced Technology, Chinese Academy of Sciences, Shenzhen, China; c University of Chinese Academy of Sciences, Beijing, China; d State Key Laboratory of Virology, Wuhan Institute of Virology, Center for Biosafety Mega-Science, Chinese Academy of Sciences, Wuhan, China; University of North Carolina at Chapel Hill

**Keywords:** Zika virus (ZIKV), capsid, tetracysteine (TC) tag, biarsenical reagents, single-particle tracking, real-time imaging

## Abstract

Real-time imaging of viruses in living cells considerably facilitates the study of virus-host interactions. However, generating a fluorescently labeled recombinant virus is challenging, especially for Zika virus (ZIKV), which causes microcephaly in neonates. The monocistronic nature of the ZIKV genome represents a major challenge for generating a replication-competent genetically engineered ZIKV suitable for real-time imaging. Here, we generated a fluorescent ZIKV by introducing the biarsenical tetracysteine (TC) tag system. After separately inserting the TC tag at six sites in the capsid protein, we found that only when we inserted the TC tag at the site of amino acids 27/28 (AA27/28, or TC27) could the genetically engineered ZIKV be rescued. Importantly, the TC27 ZIKV is characterized as replication and infection competent. After labeling the TC tag with the fluorescent biarsenical reagents, we visualized the dynamic nuclear import behavior of the capsid protein. In addition, using the single-particle tracking technology, we acquired real-time imaging evidence that ZIKV moved along the cellular filopodia and entered into the cytoplasm via endocytosis. Thus, we provide a feasible strategy to generate a replication-competent TC-tagged ZIKV for real-time imaging, which should greatly facilitate the study of ZIKV-host interactions in living cells.

**IMPORTANCE** Zika virus (ZIKV) is the mosquito-borne enveloped flavivirus that causes microcephaly in neonates. While real-time imaging plays a critical role in dissecting viral biology, no fluorescent, genetically engineered ZIKV for single-particle tracking is currently available. Here, we generated a replication-competent genetically engineered ZIKV by introducing the tetracysteine (TC) tag into its capsid protein. After labeling the TC tag with the fluorescent biarsenical reagents, we visualized the nuclear import behavior of the capsid protein and the endocytosis process of single ZIKV particle. Taken together, these results demonstrate a fluorescent labeling strategy to track the ZIKV-host interactions at both the protein level and the viral particle level. Our replication-competent TC27 ZIKV should open an avenue to study the ZIKV-host interactions and may provide applications for antiviral screening.

## INTRODUCTION

Zika virus (ZIKV) is a mosquito-borne enveloped flavivirus, and it causes severe neurological complications, especially microcephaly in neonates (1). To date, no approved vaccines nor antivirals are available against ZIKV, as the information on the ZIKV-host interactions remains limited (2, 3). Among the techniques suitable for investigating the virus-host interactions, real-time imaging serves as a potent tool that has revealed many important processes in the viral life cycle. For example, recent studies have revealed the HIV multistep infection process by tracking the fluorescently labeled virus (4–8). For real-time imaging of viruses, labeling the viral components with fluorophores is sufficient to illuminate the early infection events. However, tracking the late events (such as replication, assembly, and egress) requires genetic engineering and specific labeling of the viral protein components (9).

It is challenging to generate a replication-competent genetically engineered ZIKV for real-time imaging. The monocistronic genome of ZIKV is translated to a single polyprotein chain composed of the complete set of viral proteins (10), and the polyprotein is posttranslationally processed. Genetically engineering one viral protein may influence the translation of other viral proteins. Insertion of foreign genes into the ZIKV genome may also exert negative effects on the stability of the viral genome (11). To avoid these issues, strategies such as partial viral gene duplication and recombination-dependent lethal mutations have been recently developed for expressing heterogenous reporter genes in the context of full-length ZIKV plasmids (11, 12).

Recent studies investigating ZIKV biology by imaging have used reporters such as luciferase and green fluorescent protein (GFP). However, these reporter proteins are always designed to be cleaved off their fused proteins by a downstream 2A sequence (13, 14), in case these large-sized reporter proteins disrupt the structure of their fused proteins. The cleavage off the reporter prevents tracking either the fused viral protein or single virus particle in living cells. As an alternative to fluorescent proteins, the biarsenical tetracysteine (TC) technology represents a suitable tool for virus imaging. The TC tag is a short peptide that can be specifically labeled with membrane-permeable fluorescent biarsenical reagents (FlAsH and ReAsH) (15). The biarsenical TC tag system exhibits two advantages over fluorescent proteins. The first advantage is related to molecular size. The small size of the TC tag minimizes the risk of disrupting the structure of the fused viral protein. The second advantage is related to chromophoric efficiency. The TC tag becomes illuminated upon binding to the biarsenical reagents, while the translated fluorescent proteins require proper protein folding and chromophore maturation (16). Therefore, the biarsenical TC tag system allows successful tracking of viral proteins in living cells, including Gag and the core protein of HIV, the NS1 protein of the influenza A virus, and the μNS protein of the mammalian orthoreovirus (4, 7, 9, 17, 18).

The capsid protein of ZIKV is a structural protein that assembles the viral particle by interacting with the viral RNA and viral envelope proteins (19). In addition, the capsid protein alters host gene transcription by binding host mRNA, inhibits viral RNA silencing by interfering with Dicer, and destroys ribosome formation by interacting with ribosome biogenesis factors (20). This versatility of the capsid protein renders it a suitable candidate for fluorescence labeling. Therefore, we introduced the TC tag in the capsid protein to develop the strategy for real-time imaging of ZIKV.

## RESULTS

### Selection of TC tag insertion sites in the capsid protein.

The TC tag we used in this study is the previously optimized 12-amino-acid motif (FLNCCPGCCMEP), which shows a higher affinity for the biarsenical reagents than the original 6-amino-acid motif (CCPGCC) (21). We also added the flexible linkers GGGS and SGGG to the left and right flanks of the TC tag, respectively, to minimize the risk of disrupting the capsid protein structure. Nevertheless, it is critical to identify the optimal insertion sites to ensure that the genetically engineered ZIKV is replication competent.

**FIG 1:**
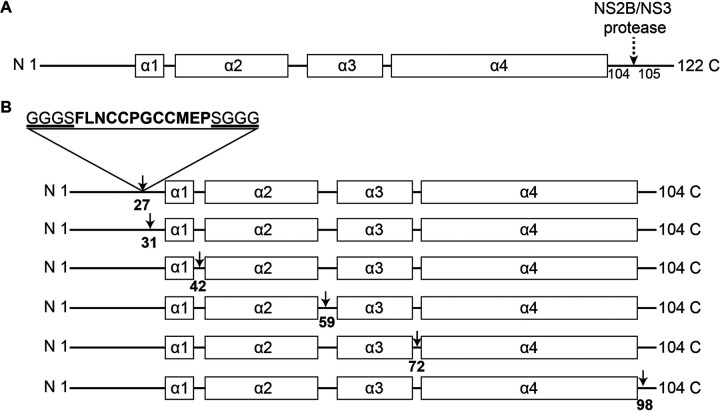
Scheme for introducing the TC tag in the capsid protein. (A) Schematic diagram of the secondary structure of the full-length capsid protein. The α-helices are shown as black boxes, and the loop/linker regions are shown as black lines. The dashed arrow indicates the viral protease NS2B/NS3 cleavage site in the capsid protein. (B) Diagram of the TC tag insertion sites in the capsid protein. Arrows indicate the selected six tetracysteine (TC) tag insertion sites. All the TC tag insertion sites are located at the loop/linker regions.

The capsid protein is translated as a peptide of 122 amino acids, consisting of a disordered N-terminal region (pre-α1 loop) and 5 α-helices. After the cleavage at the helix α4-α5 junction by the viral protease NS2B/NS3, the remaining 1 to 104 residues constitute the mature capsid protein (Fig. 1A). Therefore, we selected the TC tag insertion sites within the range of 1 to 104 residues. Since modifications in the α-helix region usually interfere with viral recovery (9), we selected the TC tag insertion sites at the loop/linker regions (Fig. 1B). At the N-terminal region, because the 1 to 25 residues are essential for the viral genome cyclization required for viral replication (14), we did not select the TC tag insertion sites within this range. At the C-terminal region, we selected the TC tag insertion sites away from the viral protease NS2B/NS3 recognition sequence. As a consequence, we selected six sites to insert the TC tag: AA27/28 (TC27), AA31/32 (TC31), AA42/43 (TC42), AA59/60 (TC59), AA72/73 (TC72), and AA98/99 (TC98) (Fig. 1B).

### Generation of the genetically engineered ZIKVs with TC-tagged capsid.

In the background of the infectious clone of the Natal-RGN (NR) strain, we replaced the nucleotide sequence between the AsiSI and SrfI restriction sites with the sequence carrying a TC tag gene inserted at one of the six selected sites. The results of our subsequent DNA sequencing indicated that we successfully constructed all six mutant plasmids. To examine whether the genetically engineered viruses could be rescued, we performed three assays: indirect immunofluorescence assay (IFA), reverse transcription (RT)-PCR analysis, and transmission electron microscopy (TEM).

**FIG 2:**
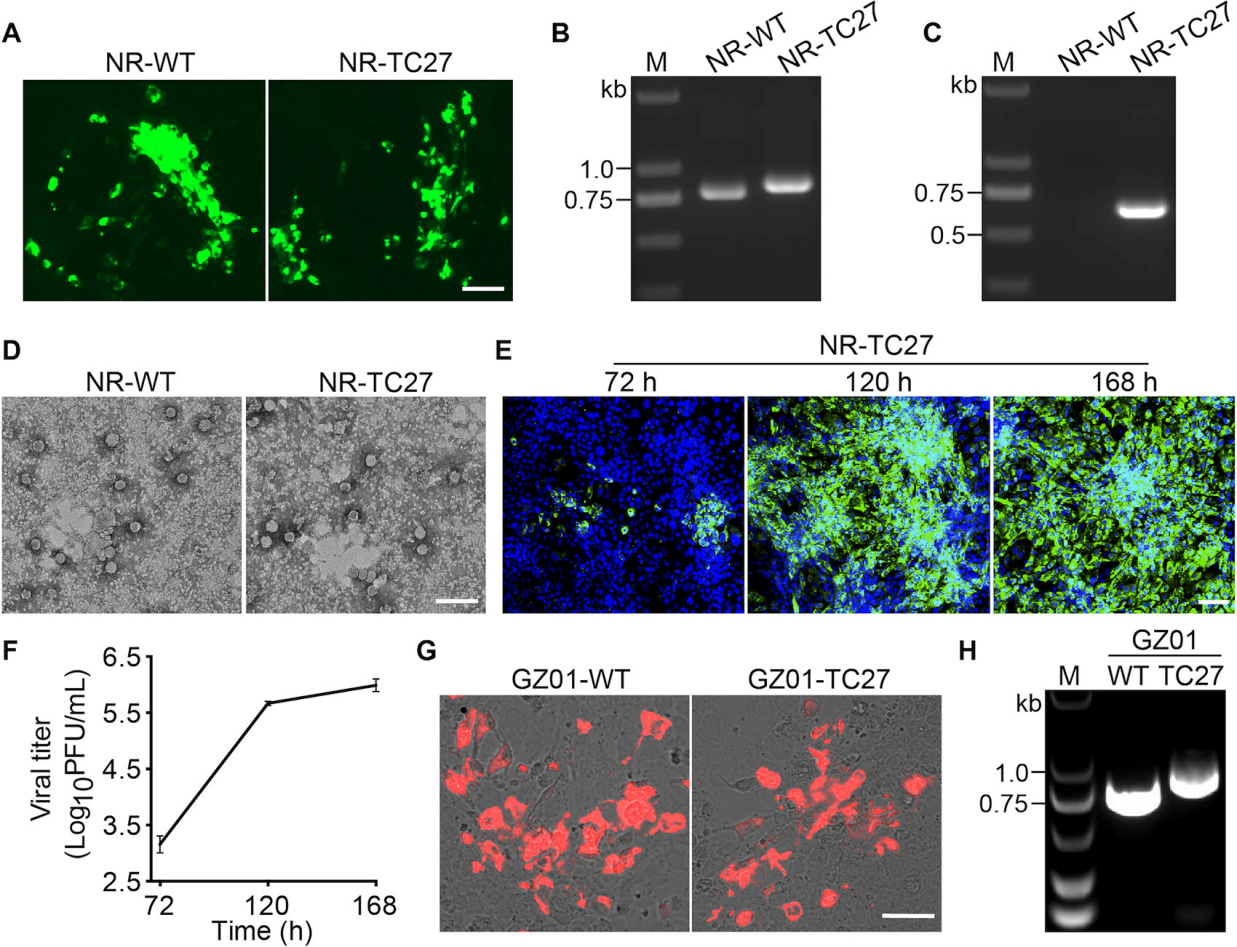
Recovery of the TC27 ZIKV. (A to E) The NR-TC27 ZIKV was rescued. (A) Immunofluorescence assay (IFA) detection of the viral capsid protein (green) expression. Bar, 50 μm. (B, C) Gel electrophoresis of the reverse transcription (RT)-PCR products amplified using the primer pair PCR-F1 and RGN verity-R (B) or the paired primers TC-specific primer and RGN verity-R (C). (D) Rescued Zika virus (ZIKV) particles observed under transmission electron microscopy (TEM). Bar, 100 nm. (E, F) Vero cells were infected with the rescued NR-TC27 ZIKV (P0) at a multiplicity of infection (MOI) of 0.005. The infected cells were fixed, and the culture supernatants were collected at 72, 120, and 168 h postinfection (hpi). The fixed cells were used to assess the viral E protein (green) expression (E), and the collected culture supernatants were used to quantify the viral titers (F). (E) Nuclei were stained with 4′,6-diamidino-2-phenylindole (DAPI) (blue). Bar, 100 μm. (F) Plaque assay was performed in three independent experiments. Error bars indicate standard deviation. (G, H) GZ01-TC27 ZIKV was rescued. (G) Expression of the viral capsid protein (red) in Vero cells. Bar, 50 μm. (H) Gel electrophoresis of the RT-PCR products using the primer pair PCR-F1 and RGN verity-R’. Lane M, DNA marker; NR, Natal-RGN; WT, wild type.

To determine whether insertion of the TC tag interferes with the capsid protein expression, we performed an IFA assay. Briefly, we separately transfected Vero cells with the wild type (WT) and six mutant plasmids and then fixed the transfected cells for immunofluorescence staining at 48 h posttransfection (hpt). As shown in Fig. 2A, only the cells transfected with either the NR-WT or NR-TC27 plasmid expressed the viral capsid protein. This result suggested that the TC-tagged capsid protein was expressed only if the TC tag was inserted at the appropriate site, and the appropriate site for the capsid protein of ZIKV to incorporate the TC tag is AA27/28.

To determine whether the rescued ZIKV could be released normally after insertion of the TC tag in the capsid protein, we collected the culture supernatants of the transfected cells and performed an RT-PCR assay. As shown in Fig. 2B, only the RT-PCR products from the culture supernatants of the cells transfected with either the NR-WT or NR-TC27 plasmid showed the band of the capsid gene. In addition, when we used the TC sequence-specific primer pair, only the RT-PCR products from the culture supernatants of the cells transfected with the NR-TC27 plasmid showed the expected TC-specific gene band (Fig. 2C). The results from DNA sequencing confirmed that the sequences of the NR-WT and NR-TC27 RT-PCR products were in full accord with their counterparts in the plasmids. Together, these results suggested that insertion of the TC tag at the site of AA27/28 did not interfere with the encapsulation of the ZIKV genome and the egress of ZIKV.

To determine whether the released ZIKVs were assembled as intact viral particles after insertion of the TC tag in the capsid protein, we collected the culture supernatants of the transfected cells and observed the rescued ZIKV particles under TEM. As shown in Fig. 2D, we observed intact viral particles in the culture supernatants of the cells transfected with either the NR-WT plasmid or the NR-TC27 plasmid. This result suggested that insertion of the TC tag at the site of AA27/28 did not interfere with the assembly of ZIKV.

Together, these results from IFA, RT-PCR, and TEM clearly demonstrated that we successfully rescued the NR-TC27 ZIKV (Passage 0 [P0]). To determine whether the generated NR-TC27 ZIKV was replication competent, we monitored the viral E protein expression and viral yields at 72, 120, and 168 h postinfection (hpi). As shown in Fig. 2E, the expression level of the viral E protein increased with time. In addition, the viral yields quantified by the plaque assay increased from 1.5 × 10^3^ PFU/mL at 72 hpi to 1.0 × 10^6^ PFU/mL at 168 hpi (Fig. 2F; Table 1). Collectively, these results showed that our generated NR-TC27 ZIKV was replication competent.

**TABLE 1:** Data set of Fig. 2F showing the multiplication kinetics of NR-TC27 ZIKV (P0)

Time	First assay (log_10_PFU/mL)			Second assay (log_10_PFU/mL)			Third assay (log_10_PFU/mL)		
72 h	3.00	3.30	3.18	3.57	4.30	3.21	2.80	3.88	3.10
120 h	5.70	5.63	5.68	5.56	5.14	5.64	4.88	5.64	5.72
168 h	5.88	6.10	6.00	5.64	6.35	6.46	5.80	5.94	6.27

To determine whether the TC27 insertion strategy applies to other clones of ZIKV strains, we constructed six mutant plasmids containing a TC tag gene at the same six sites in the background of another ZIKV clone (GZ01 strain). The results were similar, and only the TC27 ZIKV was rescued. As shown in Fig. 2G, only the cells transfected with either the GZ01-WT or GZ01-TC27 plasmid expressed the viral capsid protein. In addition, only the RT-PCR products from the culture supernatants of the cells transfected with either the GZ01-WT or GZ01-TC27 plasmid showed the band of the capsid gene (Fig. 2H). These results suggested that the TC27 insertion strategy was suitable for application to other ZIKV clones.

### Characterization of NR-TC27 ZIKV.

**FIG 3:**
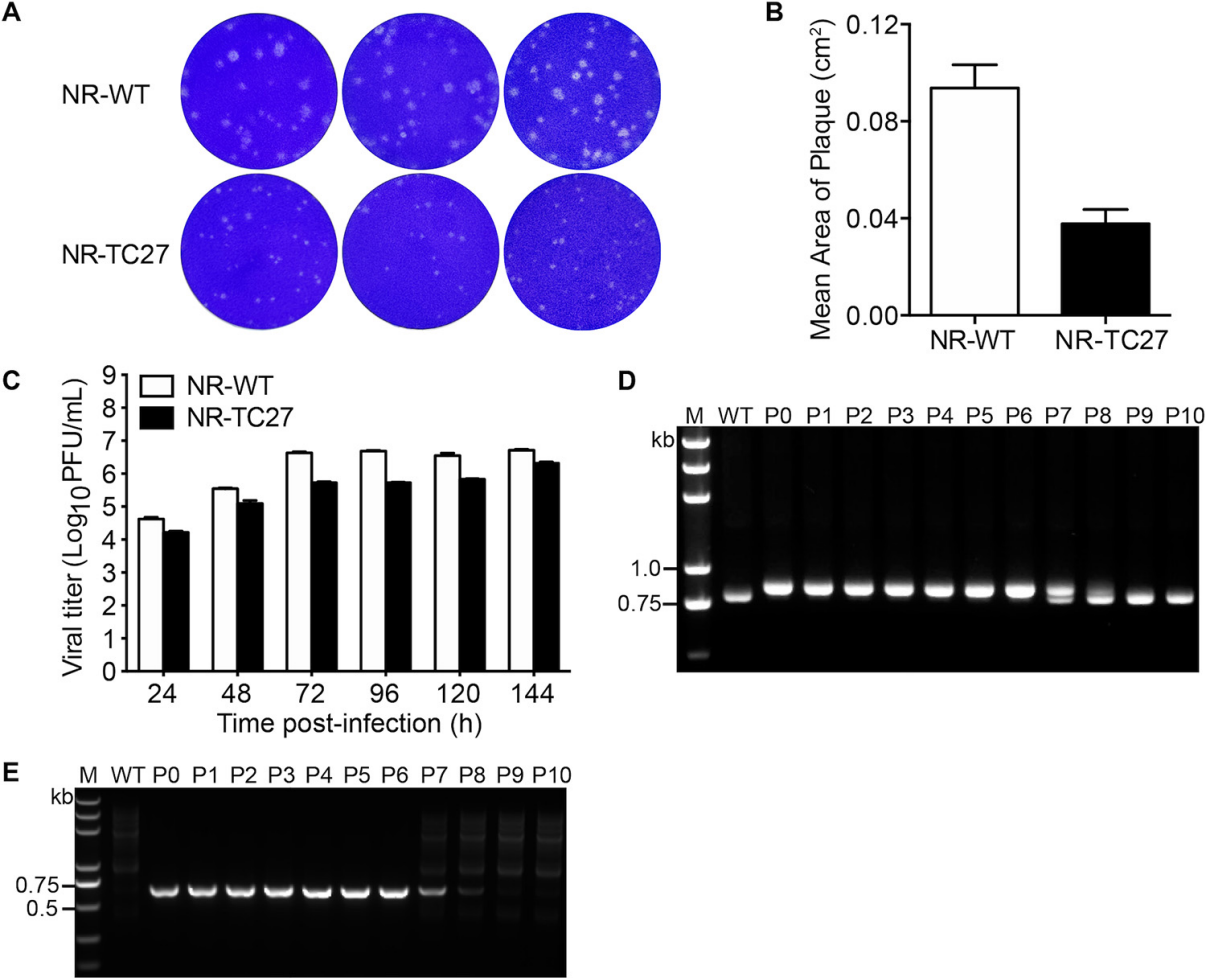
Characterization of the NR-TC27 ZIKV. (A) Plaque morphologies of the NR-WT and NR-TC27 ZIKVs (P2). Vero cells were fixed and stained with crystal violet on 3 days postinfection. (B) Comparison of plaque sizes of NR-WT and NR-TC27 ZIKV (P2). (C) Vero cells were infected with the NR-WT or NR-TC27 ZIKV at an MOI of 0.1. The culture supernatants were collected at 24, 48, 72, 96, 120, and 144 hpi. A plaque assay was used to determine the viral titer. Error bars indicate standard deviation. (D, E) Both NR-WT and NR-TC27 ZIKVs (from P0 to P10) were collected and analyzed by RT-PCR using the primer pair PCR-F1 and RGN verity-R (D) or the paired primers TC-specific primer and RGN verity-R (E). Lane M, DNA marker; P, passage.

To characterize the infectivity of the NR-TC27 ZIKV, we assessed the plaque size, as well as the replication kinetics of the NR-WT and NR-TC27 ZIKV in Vero cells. The mean plaque size of the NR-TC27 ZIKVs was 2.52 times smaller than the mean plaque size of the NR-WT ZIKVs (Fig. 3A and B). For the viral replication kinetics, we collected the culture supernatants and determined the viral titers at different time points postinfection. As shown in Fig. 3C, the growth rate of the NR-TC27 ZIKVs was slower than the growth rate of the NR-WT ZIKVs. At 144 hpi, the viral titer of the NR-TC27 ZIKVs reached 2.1 × 10^6^ PFU/mL, while the viral titer of the NR-WT ZIKVs reached 5.2 × 10^6^ PFU/mL (Fig. 3C; Table 2). Together, these results indicated that insertion of the TC tag slightly interfered with the viral infectivity and replication of ZIKV.

**TABLE 2:** Data set of Fig. 3C showing the multiplication kinetics of the NR-WT and NR-TC27 ZIKV

Assay	Time	WT (Natal-RGN) (log_10_PFU/mL)			TC27 (Natal-RGN) (log_10_PFU/mL)		
First assay	24 h	4.68	4.63	4.57	4.24	4.18	4.24
	48 h	5.57	5.53	5.54	5.00	5.18	5.10
	72 h	6.60	6.65	6.65	5.70	5.72	5.76
	96 h	6.69	6.67	6.71	5.74	5.70	5.73
	120 h	6.63	6.48	6.54	5.83	5.85	5.81
	144 h	6.74	6.71	6.70	6.33	6.27	6.35
Second assay	24 h	2.80	4.48	1.88	3.10	2.94	2.70
	48 h	5.57	5.69	5.10	4.40	4.27	4.24
	72 h	6.10	6.18	6.35	5.57	5.72	5.54
	96 h	6.68	6.54	6.57	5.74	5.70	5.73
	120 h	6.70	6.57	6.68	4.57	6.05	5.88
	144 h	7.05	7.10	7.00	6.18	6.38	6.44
Third assay	24 h	4.49	4.54	4.57	4.00	3.80	4.10
	48 h	5.40	3.18	5.49	4.70	4.64	4.73
	72 h	5.70	6.00	5.94	5.40	5.35	5.21
	96 h	6.33	6.42	6.18	4.48	5.53	5.64
	120 h	6.57	6.64	6.54	5.74	4.27	5.80
	144 h	6.14	6.88	6.80	6.40	5.38	6.72

To study the stability of the heterologous TC tag gene, we cultured the rescued NR-TC27 ZIKVs (P0) for 10 passages and then checked whether the TC tag gene was lost. As shown in Fig. 3D and E, during the first six serial passages, the bands of the TC-tagged capsid gene (Fig. 3D) and the bands of the TC-specific gene (Fig. 3E) remained identical. In addition, DNA sequencing results revealed no mutations in the TC encoding sequence in any of the first six serial passages. However, from the seventh passage, the band of the TC-tagged capsid gene was gradually converted to the band of the TC-untagged capsid gene (Fig. 3D), and the band of the TC-specific gene gradually disappeared (Fig. 3E). Further DNA sequencing results confirmed the deletion of the TC tag gene from the seventh passage. These results indicated that the exogenous TC tag gene remained stably inserted in the viral genome for the first six serial passages.

### Labeling the TC27 capsid protein in living cells.

**FIG 4:**
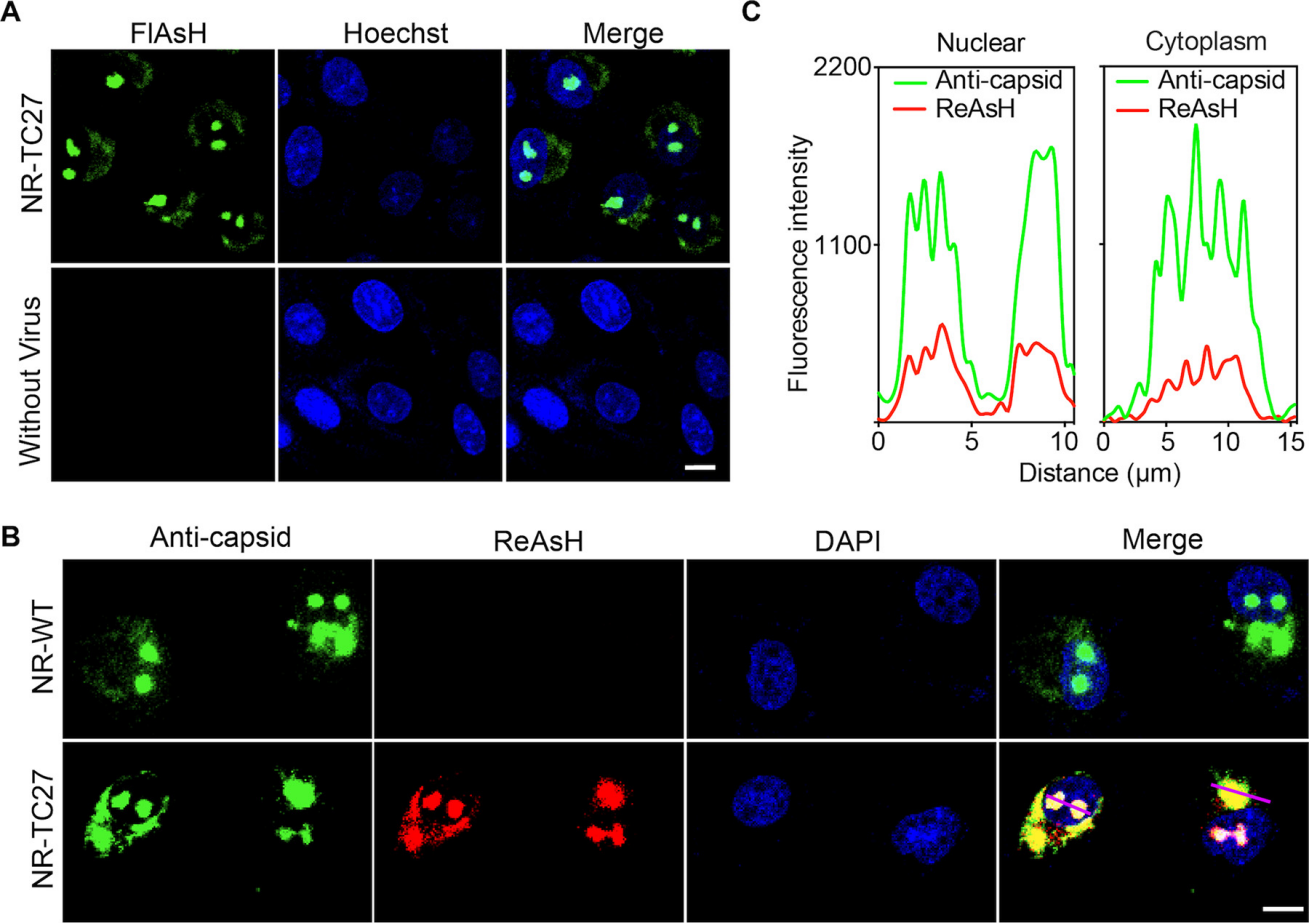
TC27 capsid protein was specifically labeled by the biarsenical reagents in living cells. (A) Vero cells were infected with/without the NR-TC27 ZIKV (P4) at an MOI of 0.1 and then stained using the biarsenical reagent FlAsH (green). Nuclei were stained with Hoechst 33258 (blue). Bar, 10 μm. (B) Vero cells were infected with either the NR-WT or NR-TC27 ZIKV (P4) at an MOI of 0.1. The infected cells were stained using both a capsid-specific antibody (green) and the biarsenical reagent ReAsH (red). Nuclei were stained with DAPI (blue). Bar, 10 μm. (C) Fluorescent signal profiles of the capsid-specific antibody and the biarsenical reagent ReAsH along the lines drawn across the cytoplasm and nucleus in panel B.

To determine whether the TC tag can be labeled following fusion with the capsid protein, we infected Vero cells with/without the NR-TC27 ZIKV and stained these cells using the biarsenical reagent FlAsH. As shown in Fig. 4A, strong biarsenical fluorescent signals were detected in the infected cells, while no biarsenical fluorescent signal was detected in the uninfected cells. This result clearly showed that the biarsenical reagent FlAsH only labeled the viral components of NR-TC27 ZIKV, without nonspecific labeling of the cellular components.

To confirm that the biarsenical reagent-labeled protein represented the TC27 capsid protein, we performed an IFA assay using a capsid-specific antibody after labeling the infected cells with ReAsH. As shown in Fig. 4B, the cells infected with the NR-WT ZIKV were labeled only by the capsid-specific antibody, while the cells infected with the NR-TC27 ZIKV were labeled by both the capsid-specific antibody and the biarsenical reagent ReAsH (Fig. 4B). Moreover, we found that the fluorescent signals of the capsid-specific antibody and the biarsenical reagent ReAsH largely overlapped (Mander’s correlation coefficient: 0.909 ± 0.038, *n* = 24) and that the two fluorescent signals changed in a similar pattern along the lines drawn across the cytoplasm and nucleus (Fig. 4C). Together, these results demonstrated that the biarsenical reagent-labeled protein was the TC27 capsid protein. In addition, we noticed that the TC27 capsid proteins, just like the WT capsid proteins, were expressed and distributed at nucleoli and the perinuclear regions (Fig. 4B), which suggested that insertion of the TC tag exerted no negative effects on the expression and subcellular localization of the capsid protein.

### Tracking the TC27 capsid protein in living cells.

**FIG 5:**
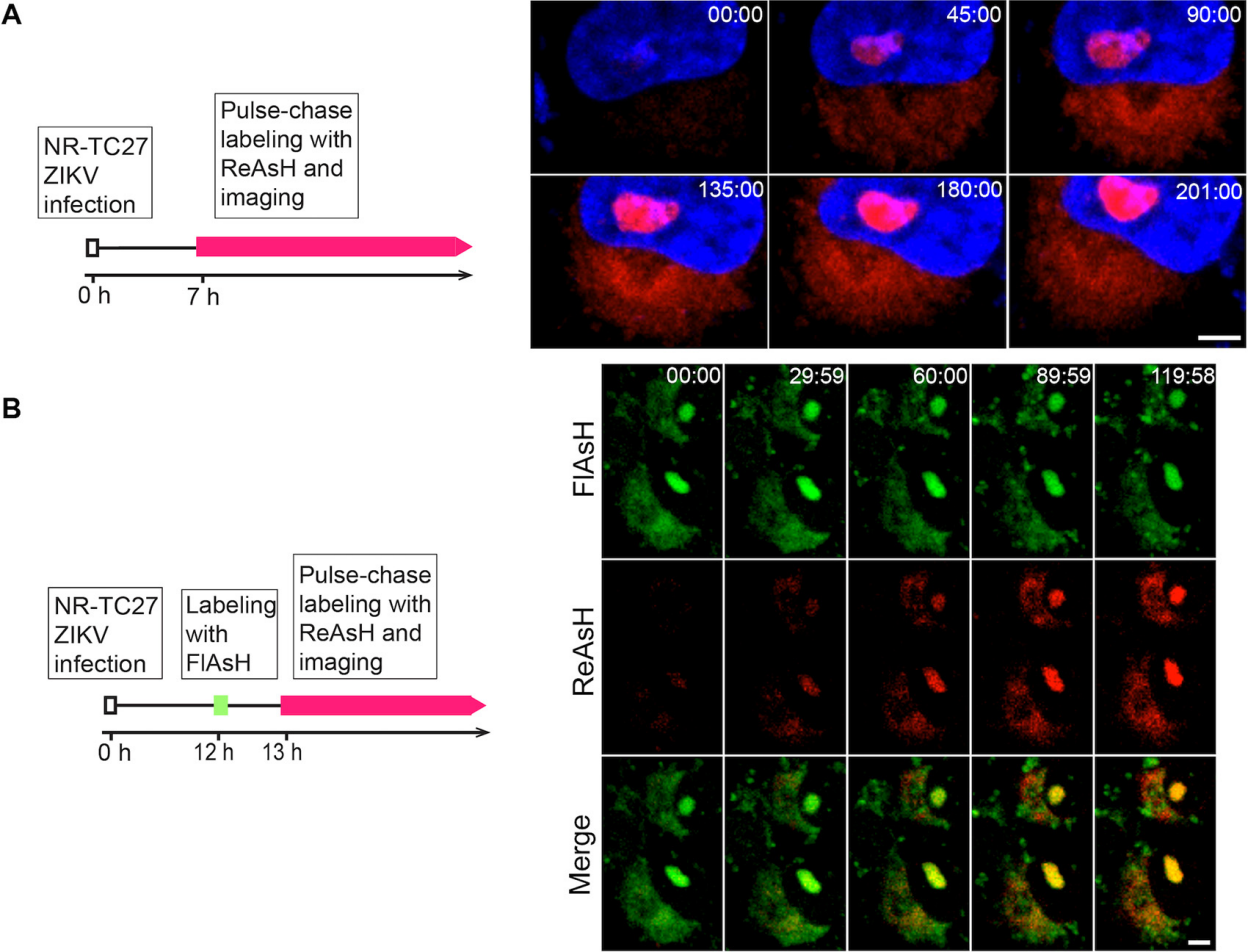
Real-time imaging of the TC27 capsid protein in living cells. (A) Real-time imaging of the expression and nuclear import behavior of the TC27 capsid protein in living cells. Schematic design for tracking the TC27 capsid protein in living cells (left). Vero cells were infected with the NR-TC27 ZIKV (P4) at an MOI of 1 and then pulse-chase labeled by ReAsH (red) at 7 hpi. Time-lapse images were captured for 201 min (right). Nuclei were stained with Hoechst 33258 (blue). Bar, 5 μm. (B) Real-time imaging of the TC27 capsid proteins expressed at different time in living cells. Schematic design for tracking the TC27 capsid proteins expressed at different time (left). Vero cells were infected with the NR-TC27 ZIKV (P4) at an MOI of 1. The previously expressed TC27 capsid proteins were labeled with FlAsH (green), and the newly expressed TC27 capsid proteins were pulse-chase labeled with ReAsH (red). The imaging time was 120 min. Bar, 5 μm.

After specifically labeling the TC27 capsid protein in living cells, we were able to track the intracellular behavior of the viral capsid protein. We infected Vero cells with the NR-TC27 ZIKV and replaced the culture supernatant with 500 μL of Opti-MEM containing ReAsH (0.4 μM) at 7 hpi to pulse-chase label the intracellular TC27 capsid protein (Fig. 5A, left). The time-lapse images were captured every 2 min for 201 min. As shown in Fig. 5A, the ReAsH fluorescent signal at nucleoli and the perinuclear regions increased with time (Fig. 5A, right; Movie S1), depicting the expression and nuclear import process of the TC27 capsid protein. In addition, we noticed that, concomitantly with entry of viral capsid protein into the nucleus, the nuclear morphology gradually turned concave (Fig. 5A, right; Movie S1).

To study whether the behaviors and subcellular distribution of the capsid proteins are affected by their expression time, we labeled the TC27 capsid proteins expressed at different times with different biarsenical reagents. Briefly, we used the first biarsenical reagent FlAsH (green) to label the previously expressed TC27 capsid proteins at 12 hpi and then used the second biarsenical reagent ReAsH (red) to pulse-chase label the newly expressed TC27 capsid proteins at 13 hpi (Fig. 5B, left). We found that the previously expressed TC27 capsid proteins (green) were distributed expectedly at nucleoli and the perinuclear regions. Likewise, the newly expressed TC27 capsid proteins (red) gradually appeared and were distributed at nucleoli and the perinuclear regions (Fig. 5B, right; Movie S2). Together, these results indicated that the behaviors and subcellular distribution of the capsid proteins were not affected by their expression time.

### Real-time imaging of the infection process of single NR-TC27 ZIKV particle.

**FIG 6:**
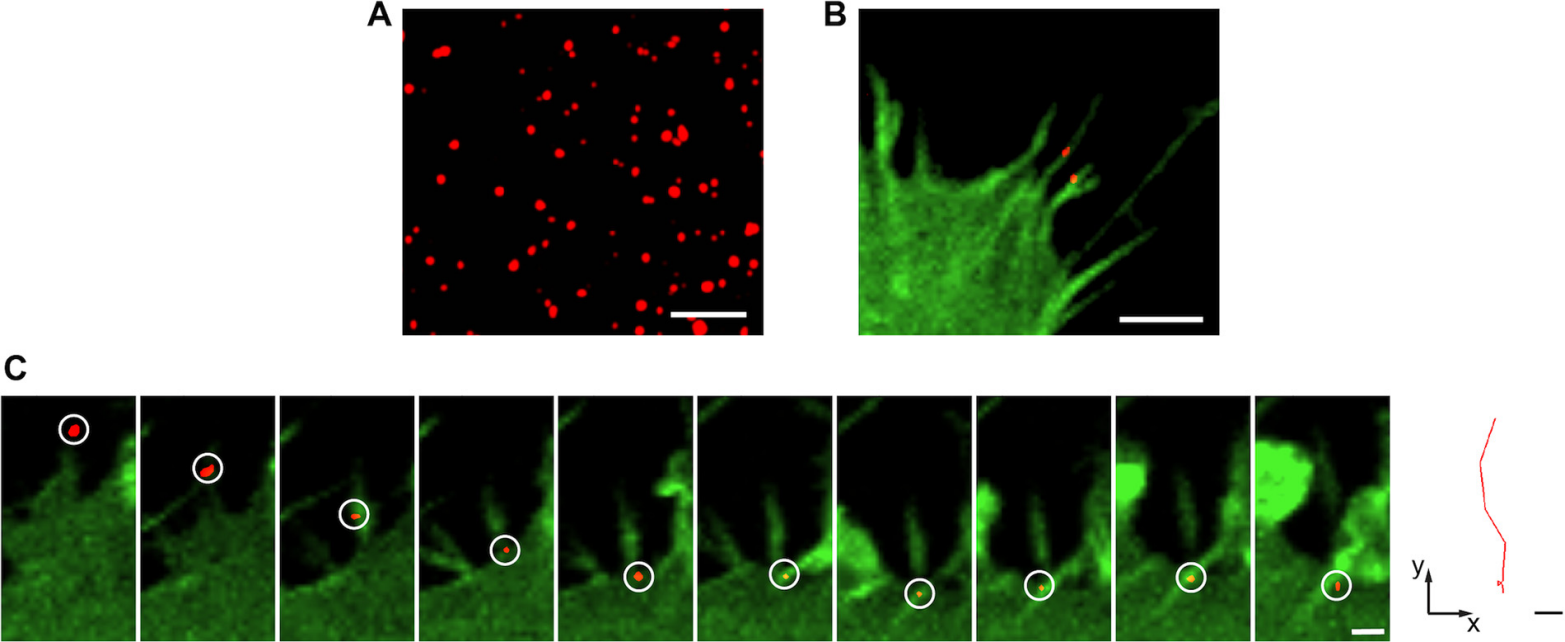
ZIKV bound and moved along cellular filopodia before cell entry. (A) The ReAsH-labeled NR-TC27 ZIKV (P3) particles (red) observed using confocal microscopy. Bar, 5 μm. (B) The NR-TC27 ZIKV particles (red) bound on the cellular filopodia of the hCMEC/D3^Lifeact-EGFP^ cells (green). Bar, 5 μm. (C) Time-lapse images of single NR-TC27 ZIKV particle moving along cellular filopodia (left; bar, 2 μm) and the corresponding trajectory (right; bar, 2 μm).

As the structure of mature viral particle is compactly organized, we wondered whether we could label and track the packaged NR-TC27 ZIKV particle. Therefore, we directly labeled the NR-TC27 ZIKV particles using the biarsenical reagent ReAsH (red) and observed them under a laser scanning confocal microscope. As shown in Fig. 6A, the NR-TC27 ZIKV particles were observed as single red dots, indicating that we successfully labeled the packaged NR-TC27 ZIKV particles with the biarsenical reagent.

ZIKV has the ability to cause fetal microcephaly; however, its precise neuro-invasive mechanism remains to be elucidated. As ZIKV needs to traverse the blood-brain barrier (BBB) to infect the central nervous system, we used the human cerebral microvascular endothelial cell line (hCMEC/D3), a model BBB cell line, to study the cell infection process of single ZIKV particle.

Before cell entry, viruses can make use of finger-like cellular protrusions called filopodia to reach the cell body (22). To explore whether ZIKV binds on cellular filopodia before cell entry, we first generated a genetically engineered hCMEC/D3 cell line, in which the fluorescent Lifeact-EGFP was stably overexpressed, to visualize cellular filopodia. We then infected the hCMEC/D3^Lifeact-EGFP^ cells using the ReAsH-labeled NR-TC27 ZIKVs and tracked the infection process using confocal microscopy. As shown in Fig. 6B, the NR-TC27 ZIKV particles attached to the filopodia of the hCMEC/D3^Lifeact-EGFP^ cells were clearly visible. Moreover, the time-lapse images showed that ZIKV moved along filopodia before cell entry (Fig. 6C; Movie S3). The moving trajectory is shown in the right panel of Fig. 6C.

**FIG 7:**
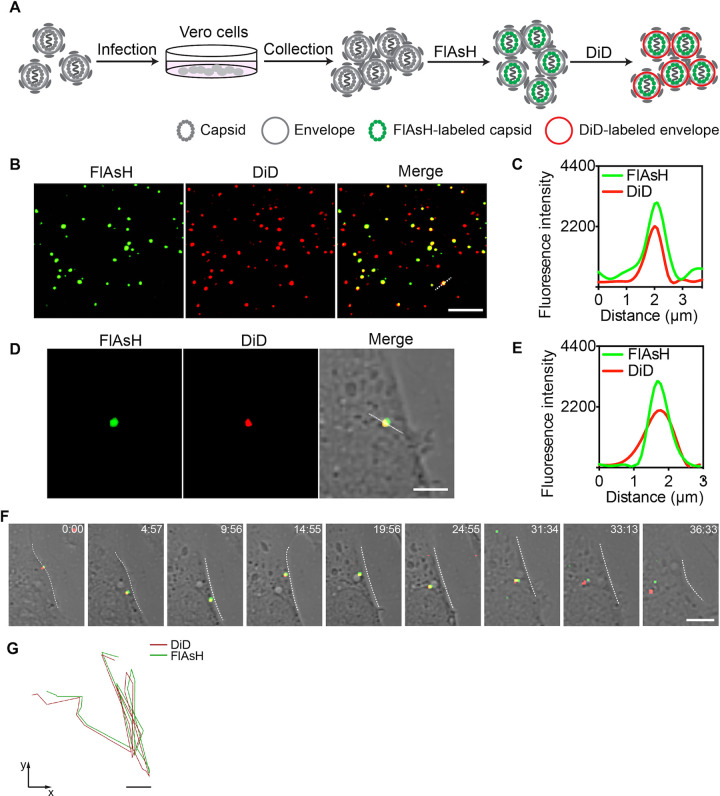
Double-labeling and tracking NR-TC27 ZIKV (P3) particles. (A) Schematic design for double-labeling the NR-TC27 ZIKV particles with FlAsH and DiD. (B) Double-labeled NR-TC27 ZIKV particles observed using confocal microscopy. Bar, 5 μm. (C) Fluorescent signal profiles of FlAsH and DiD along the dashed lines drawn in panel B. (D) The single NR-TC27 ZIKV particle we tracked in panel F was double-labeled. Bar, 2 μm. (E) Fluorescent signal profiles of FlAsH and DiD along the dashed lines drawn in panel D. (F) Time-lapse images of the dynamic internalization process of the single double-labeled NR-TC27 ZIKV particle. Bar, 2 μm. (G) The corresponding trajectories of the green FlAsH and red DiD on the single NR-TC27 ZIKV particle. Bar, 2 μm.

Internalization of enveloped viruses into cells is mainly mediated via two pathways: endocytosis and membrane fusion. To explore how ZIKV is internalized into cells, we first double-labeled the NR-TC27 ZIKV particles. As shown in Fig. 7A, we stained the TC27 capsid protein using the biarsenical reagent FlAsH (green) and then stained the viral envelope using a lipophilic reagent DiD (red). Subsequently, we observed these stained NR-TC27 ZIKV particles using confocal microscopy to check whether they were successfully double-labeled. As shown in Fig. 7B, many green FlAsH-labeled NR-TC27 ZIKV dots merged with the red DiD-labeled NR-TC27 ZIKV dots (Mander’s correlation coefficient: 0.94 ± 0.04, *n* = 100). In addition, the fluorescent signals of FlAsH and DiD changed in a similar pattern along the dashed line drawn across a ZIKV dot (Fig. 7C). Together, these results clearly demonstrated that we successfully double-labeled the NR-TC27 ZIKV particles. We next used these double-labeled NR-TC27 ZIKVs to infect the hCMEC/D3^Lifeact-EGFP^ cells and tracked the dynamic infection process of single ZIKV particle for 37 min. As shown in Fig. 7D and E, the single NR-TC27 ZIKV particle we tracked was double-labeled, since the fluorescent signals of FlAsH and DiD overlapped and changed in a similar pattern along a dashed line. The time-lapse images shown in Fig. 7F reveal the dynamic internalization process of the single ZIKV particle. Briefly, the single ZIKV particle bound on the cell surface, then entered the cell and moved around near the membrane for 20 min, and finally moved toward the cytoplasm interior (Fig. 7F; Movie S4). The moving trajectory is shown in Fig. 7G. During the internalization process, we noticed that the DiD-labeled envelope did not separate from the FlAsH-labeled TC27 capsid protein. This result indicated that ZIKV entered into the hCMEC/D3 cells via endocytosis instead of membrane fusion. After the process of endocytosis, we observed the dissociation of the capsid protein from the viral envelope at the latter stage when the double-labeled ZIKV particle traveled toward the cytoplasm interior (Fig. 7F and G; Movie S5).

## DISCUSSION

In this study, we developed a strategy to generate a replication-competent TC-tagged ZIKV, which can be tracked in living cells in real time. The TC tag was inserted at the site of AA27/28 in the capsid protein. After labeling the TC tag with the fluorescent biarsenical reagents, we visualized the dynamic nuclear import behavior of the capsid protein and the endocytosis process of a single ZIKV particle.

Genetic engineering of a viral protein often affects the expression, function, and intracellular trafficking of the recombinant protein and the rescue, replication, and infection competence of the recombinant virus (23–25). Engineering the monocistronic genome of ZIKV is more complex (11), particularly the capsid protein, which is the first protein to be translated in the viral polyprotein chain and plays critical roles in viral assembly (26–28). Therefore, it is challenging to identify a site in the capsid protein that is suitable to incorporate the foreign TC tag. We tested six sites in the loop/linker regions (Fig. 1B) and found that only when the TC tag was inserted at the site of AA27/28 in the pre-α1 loop could the genetically engineered ZIKV be rescued (Fig. 2). When we inserted the TC tag at any of the remaining five sites, the capsid protein was not expressed. We speculated that insertion of the TC tag gene at the five inappropriate sites disturbed the translation of the capsid protein, as the capsid-encoding sequence contains many *cis*-acting elements (29, 30). It was reported that the α-helix region is not suitable for genetic engineering (9). Here, we showed that the loop/linker regions in the capsid protein of ZIKV are also sensitive for genetic modifications. All these difficulties in genetic engineering prevent generation of a replication-competent mutant ZIKV for real-time imaging. Generally, the heterogenous reporter genes inserted in viral genome are susceptible to deletion and mutation (31). In the genome of NR-TC27 ZIKV, the exogenous TC tag gene remained stably inserted until the seventh passage (Fig. 3D and E), indicating that the rescued NR-TC27 ZIKV was reliable to be used within the first six serial passages. As the rescued NR-TC27 ZIKV was replication competent (Fig. 2E and F), we can use it to investigate the ZIKV-host interactions in the whole viral life cycle. The reason why the viral infectivity of NR-TC27 ZIKV was slightly attenuated (Fig. 3A to C) may be that insertion of the TC tag caused the capsid protein intrinsic disorder (PID), as the PID levels of flavivirus capsid are positively correlated with the viral virulence (32).

After labeling the TC27 capsid protein with the fluorescent biarsenical reagents, we visualized the dynamic nuclear import behavior of the capsid protein (Fig. 5A). As the nucleolar localized capsid protein is involved in nucleosome formation, ribosomal stress, and Tp53-mediated neuronal apoptosis (33–35) via currently unclear mechanisms, real-time imaging of the capsid protein in living cells, as we achieved here, should contribute to unveiling these important questions. When we labeled the TC27 capsid proteins expressed at different time with different biarsenical reagents, we found that the behaviors and subcellular distribution of the TC27 capsid proteins were unaffected by their expression time (Fig. 5B), suggesting that the functions of the capsid protein were independent of the expression time. Nevertheless, differentially labeling the TC-tagged proteins expressed at different time is a useful method to track their different intracellular behaviors.

Labeling TC-tagged virus is more challenging than labeling TC-tagged protein, as the viral structure is compactly organized. For example, the TC-tagged core protein of hepatitis C virus (HCV) was reliably imaged for investigating the intracellular trafficking of the viral core protein; however, the extracellular HCV particles failed to be labeled (36). This may be the reason why we successfully labeled most TC27 capsid proteins (Fig. 4B) but labeled only some NR-TC27 ZIKV particles (Fig. 7B). In addition, nonspecifically labeling the outer envelope with DiD is easier than specifically labeling the inner well assembled capsid protein with FlAsH, so we successfully labeled most TC27 ZIKV particles with DiD but only labeled a part of TC27 ZIKV particles with FlAsH (Fig. 7B). The membrane-permeable property of the fluorescent biarsenical reagents may contribute to the successful labeling of NR-TC27 ZIKV, given that the pre-α1 loop where the TC tag was incorporated was shown embedded in the lipid bilayer (19).

Previously, the cell infection mechanisms of ZIKV were mainly addressed indirectly by using blocking agents such as chloroquine, dynasore, chlorpromazine, and genistein (37–39). Here, we use the labeled NR-TC27 ZIKV to directly investigate the cell infection mechanism of ZIKV, with the help of a single-particle tracking technique. Before internalization, viruses usually hijack cellular filopodia to reach the cell body (40). We found that ZIKV also bound and moved along cellular filopodia before cell entry (Fig. 6B and C). After labeling both the capsid protein and the envelope of NR-TC27 ZIKV, we acquired real-time imaging evidence that ZIKV was internalized into the hCMEC/D3 cells via endocytosis (Fig. 7F). This piece of evidence is in agreement with a recent study that found that the process of ZIKV crossing the hCMEC/D3 cell-monolayer was blocked by pharmacological inhibitors of endocytosis (41). Recent studies found that ZIKV establishes persistent infection in the BBB model cell line hCMEC/D3 (42). Interestingly, ZIKV infection fails to damage the hCMEC/D3 cells (42, 43). Therefore, BBB may act as a viral reservoir for ZIKV and provide a protective niche that fosters viral spread to the brain (43). The mechanism through which ZIKV balances BBB cell survival and viral replication remains to be clarified. As we achieved imaging ZIKV at both the protein level and the viral particle level, our TC27 ZIKV should facilitate investigating this important issue.

To our best knowledge, this is the first report of successful application of the biarsenical tetracysteine technology in ZIKV research. As the capsid protein interacts with a larger scope of targets in the host cells than the other viral proteins of ZIKV, our real-time imaging of the capsid protein should greatly facilitate investigating these complex ZIKV-host cell interactions (20). In principle, the strategy we developed here for real-time imaging of ZIKV should be suitable for other flaviviruses, since the charge distribution, physicochemical properties, and structure of the flavivirus capsid are well conserved (20, 44). As reporter-expressing viruses are valuable tools for high-throughput antiviral screening (45), our fluorescent TC27 ZIKV should also advance the development of such drug screening applications.

## MATERIALS AND METHODS

### Cells and antibodies.

Vero cells were cultured at 37°C in Dulbecco’s modified Eagle’s medium (DMEM) (Invitrogen) supplemented with 10% fetal bovine serum (FBS) (Invitrogen) and 1% penicillin-streptomycin (Invitrogen). A human brain capillary endothelial cell line (hCMEC/D3) was cultured in EGM-2 MV microvascular endothelial cell growth medium bullet kit (Lonza, catalog No. CC-3202) containing 5% FBS, 0.04% hydrocortisone, 0.4% human fibroblast growth factor B (hFGF-B), 0.1% vascular endothelial growth factor (VEGF), 0.1% long(R3)-insulin-like growth factor-1 (R3-IGF-1), 0.1% ascorbic acid, 0.1% human epidermal growth factor (hEGF), and 0.1% gentamicin sulfate amphotericin B (GA-1000).

The antibody for Zika capsid (GeneTex, catalog No. GTX133317) is a rabbit polyclonal antibody that recognizes the full-length Zika capsid recombinant protein. The 4G2 antibody (Millipore, catalog No. MAB10216) is a mouse monoclonal antibody that recognizes the fusion loop at the extremity of domain II of the flavivirus E protein. Alexa Fluor 488- and Alexa Fluor 561-conjugated secondary antibodies were obtained from Invitrogen (Thermo Fisher Scientific, China).

### Plasmid construction.

The details of the full-length cDNA plasmid pACYC177-Natal-RGN have been reported previously (46). The unique restriction endonuclease sites of AsiSI and SrfI, which are located upstream and downstream of the capsid-encoding sequence, were chosen for the replacement of the mutant sequences. The sequence between the AsiSI and SrfI restriction sites was divided into three fragments: fragment A, the sequence before the capsid-encoding sequence; fragment B, the capsid-encoding sequence; and fragment C, the sequence after the capsid-encoding sequence. The mutant capsid-encoding segments carrying a TC encoding sequence shown in Fig. 1B were synthesized by Shanghai Generay Biotech Co., Ltd. Primers were designed to ensure that fragments A and C shared a homologous sequence with the plasmid backbone. Fragment A was amplified with the primer pair of RGN-1 and RGN-2, and fragment C was amplified with the primer pair of RGN-5 and RGN-6. The plasmid pACYC177-Natal-RGN was used as the template to amplify both fragments A and C. The mutant fragment B was obtained by amplifying the synthesized mutant capsid-encoding segments with the primer pair of RGN-3 and RGN-4. The PCR products of fragments A and C and mutant fragment B were then fused with the linearized vectors by homologous recombination using the pEASY-Basic seamless cloning and assembly kit according to the manufacturer’s instructions (TransGen Biotech). The primer sequences are listed in Table 3.

**TABLE 3:** The primers for pACYC177-TC-Natal-RGN vectors

Primer	Sequence (5′ to 3′)
RGN-1	AATTGTCCTTTTAACAGCGATCGC
RGN-2	CCTCCGGATTTCTTTTTTGGGTT
RGN-3	ATGAAAAACCCAAAAAAGAAATCCGG
RGN-4	TGCCATAGCTGTGGTCAGC
RGN-5	TGCTGACCACAGCTATGGCAG
RGN-6	ACGTGGACCTTAGTGCCCGGGC
PCR-F1	AGTTGTTGATCTGTGTGAATCAGAC
TC specificity-F	GAACTGCTGCCCCGGCTGCTG
RGN verity-R	AGCTTCCTAGTGGAATGGGAGG

The details of the infectious clone of pACNR-GZ01-Intron-IC have been reported previously (47). The mutant plasmids were constructed by overlapping PCR, and the PCR products were cloned into the pACNR-GZ01-Intron-IC plasmid between the NotI and KpnI restriction sites. Before *in vitro* transcription, the linearized plasmids were digested with the restriction enzyme XhoI, cleaned up with phenol-chloroform and chloroform, and then precipitated by ethanol. The RNA transcripts were generated using the RiboMAX large-scale RNA production system SP6 kit (Promega) according to the manufacturer’s instructions. In brief, a total of 30 μL reaction mixture was prepared as follows: 6 μL SP6 transcription 5× buffer, 6 μL rNTPs (25 mM ATP, CTP, GTP, 3 mM UTP), 13 μL linear DNA template (2 μg total) plus nuclease-free water, and 3 μL enzyme mix (SP6). After incubation for 3.5 h at 37°C, the DNA templates were removed, and the RNA transcripts were purified and spliced. The primer sequences are listed in Table 4.

**TABLE 4:** The primers for pACNR-TC-GZ01-Intron-IC vectors

Primer name	Sequence (5′ to 3′)
Primer 1	TAAACAAATAGGGGTTCCGCG
TC(27)-1	CCATACAACAGCCAGGACAACAATTGAGAAAGCTGCCGCCGCCAAAGGGGCTCACACGGGCT
TC(27)-2	GTTGTCCTGGCTGTTGTATGGAACCTAGCGGCGGCGGCGGAGGCTTGAAGAGGCTGC
TC(31)-1	CCATACAACAGCCAGGACAACAATTGAGAAAGCTGCCGCCGCCCTTCAAGCCTCCAAAGGGGC
TC(31)-2	GTTGTCCTGGCTGTTGTATGGAACCTAGCGGCGGCGGCAGGCTGCCAGCCGGACTTCTG
TC(42)-1	CCATACAACAGCCAGGACAACAATTGAGAAAGCTGCCGCCGCCCCCATGACCCAGCAGAAGTC
TC(42)-2	GTTGTCCTGGCTGTTGTATGGAACCTAGCGGCGGCGGCCCCATCAGGATGGTCTTGGC
TC(59)-1	CCATACAACAGCCAGGACAACAATTGAGAAAGCTGCCGCCGCCGATTGCCGTGAATCTCAAGAAG
TC(59)-2	GTTGTCCTGGCTGTTGTATGGAACCTAGCGGCGGCGGCAAGCCATCACTGGGTCTCAT
TC(72)-1	CCATACAACAGCCAGGACAACAATTGAGAAAGCTGCCGCCGCCCACTGAACCCCATCTATTGATGAG
TC(72)-2	GTTGTCCTGGCTGTTGTATGGAACCTAGCGGCGGCGGCGGGAAAAAAGAGGCTATGGAAAT
TC(98)-1	CCATACAACAGCCAGGACAACAATTGAGAAAGCTGCCGCCGCCCCTAGCATTGATTATTCTCAGCA
TC(98)-2	GTTGTCCTGGCTGTTGTATGGAACCTAGCGGCGGCGGCAAGGAGAAGAAGAGACGAGGC
Primer 2	GGTACCGCATCTCGTCTCC
RGN verity-R′	AGCTTCCTAGTGGAATGGGAAGG

All plasmid transformations were performed using the Escherichia coli strain TOP10. The purified plasmids were fully sequenced to ensure that the used plasmids carried no undesired mutations.

### Transfection.

The plasmids were transferred into Vero cells using the Lonza Nucleofector 2b device as the Amaxa cell line Nucleofector kit V instructs. Briefly, Vero cells seeded in a dish were harvested by trypsinization and centrifugation at 90 × *g* for 10 min. The obtained cells were resuspended with the premixed Nucleofector solution (82 μL Nucleofector solution V and 18 μL supplemental buffer) and then mixed with 10 μg of the plasmid. A volume of 100 μL cell mixture was transferred to a cuvette followed by electroporation with Nucleofector program V-001. Next, fresh culture medium was added into the cuvette immediately, and the cells were gently transferred to the prepared 6-well plate. The supernatants were removed 6 hpt, and the cells were washed three times in phosphate-buffered saline (PBS) buffer. Subsequently, fresh culture medium was added to culture the cells at 37°C. After 48 h of culturing, the culture supernatants were collected and stored in aliquots at −80°C.

### RNA extraction and RT-PCR assay.

The genomic RNA of the WT and genetically engineered ZIKV were extracted using the PureLink viral RNA/DNA minikit (Invitrogen). The first cDNA strand was synthesized with the TransScript one-step gDNA removal and cDNA synthesis SuperMix (TransGen Biotech). The reaction mixture was prepared as follow: 7 μL total RNA/mRNA, 1 μL random primer (0.1 μg/μL), 10 μL 2× TS reaction mix, 1 μL TransScript RT/RI enzyme mix, and 1 μL gDNA remover. Next, the mixture was incubated at 25°C for 10 min, 42°C for 30 min, and 85°C for 5 s and placed on ice for 2 min. Using the synthesized cDNA as the template, the target sequence was amplified.

### Indirect immunofluorescence assay.

At the indicated time points, the culture supernatants were removed, and the cells were rinsed in cold PBS and fixed with cold 4% paraformaldehyde for 30 min. After being washed three times in PBS buffer, the cells were permeabilized with 0.5% Triton X-100 for 20 min at room temperature and then washed again three times (each wash for 5 min). Subsequently, the cells were incubated with blocking buffer containing 5% goat serum for 1 h at 37°C and then exposed to the primary antibody diluted with 1% goat serum (1:1,000) at 37°C for 1.5 h, followed by five wash steps in PBS buffer containing 0.05% Tween-20 (each wash for 5 min). The cells were then incubated with Alexa Fluor 488/Alexa Fluor 568-conjugated secondary antibodies diluted with 1% goat serum (1:1,000) at 37°C for 1 h. After washing, the cells were stained with 4′,6-diamidino-2-phenylindole (DAPI) and observed under a fluorescence microscope.

### Transmission electron microscopy (TEM).

Concentrated ZIKV particles were absorbed onto a carbon-coated copper grid for 1 to 2 min. After negative staining with uranyl acetate, the grids were examined using TEM (Tecnai Spirit, 100 KV) at a magnification of ×98,000.

### ZIKV propagation and stock preparation.

For all assays, the used ZIKVs were propagated in Vero cells. Vero cells were cultured in DMEM (10% FBS) for 2 to 3 days. When the cell confluence reached 90 to 95%, the viral inoculum was added. After incubation for 2 h with gentle shaking every 15 min, the cells were washed once in PBS buffer and then cultured in fresh DMEM (2% FBS) medium for another 5 to 6 days. When the cytopathic effect (CPE) was progressed through 70 to 80%, the culture supernatants were collected by centrifugation at 1,300 × *g* for 10 min at 4°C. The culture supernatants were aliquoted and stored at −80°C until further use.

### Plaque assay.

When Vero cells preseeded in 6-well plates reached 70 to 80% confluence, they were infected with 800 μL of ZIKV inoculum, which was 10-fold diluted serially in DMEM (2% FBS). Subsequently, the infected cells were incubated for 2 h at 37°C, with shaking every 15 min. At the end of the absorption step, the viral inoculum was discarded, and 2 mL of DMEM/agarose (vol/vol 1:1) containing 2% FBS and 1% penicillin/streptomycin was overlaid to each well. After 4 to 5 days of incubation, the cells were fixed with 4% paraformaldehyde for 30 min at room temperature. The agarose was removed, and the cells were stained with crystal violet for 10 min. After removal of crystal violet, the cells were washed a number of times with tap water. The plates were dried at room temperature, and the number of plaques for each replicate was counted.

### Viral multiplication kinetics.

Vero cells preseeded in 12-well plates were infected with the NR-WT or NR-TC27 ZIKV at an multiplicity of infection (MOI) of 0.1. After incubation at 37°C for 2 h with gentle shaking every 15 min, the culture supernatants were removed, and the cells were washed twice in PBS buffer. Next, 1 mL of DMEM (2% FBS) was added to each well. The culture supernatants were harvested daily for 6 consecutive days and stored at −80°C. The culture supernatants were not thawed before titer determination.

### Biarsenical reagents labeling.

Vero cells preseeded on 20-mm glass coverslips were infected with/without the NR-WT or NR-TC27 ZIKV at an MOI of 0.1. At the indicated time points, the culture supernatants were removed, and the cells were rinsed once in Opti-MEM medium (Invitrogen). For FlAsH/ReAsH (Invitrogen) labeling, the cells were incubated in 500 μL of labeling medium containing 2.5 μM FlAsH/ReAsH for 10 min at 37°C and were washed three times in Opti-MEM medium containing 5 × BAL (British anti-Lewisite) (each wash for 5 min). Subsequently, the cells were stained with Hoechst 33258 or fixed for IFA.

For FlAsH/ReAsH pulse-chase labeling, the infected cells were cultured in Opti-MEM containing 0.4 μM FlAsH/ReAsH. For live-cell imaging, the cell nuclei were stained using Hoechst 33258 for 10 min at 37°C and washed three times in Opti-MEM medium.

### ZIKVs purification and fluorescent labeling.

The culture supernatants harvested above were centrifuged at 10,414 × *g* for 50 min at 4°C to remove cell debris and then filtered through a 0.45-μm membrane (Millipore). The ZIKV particles were precipitated with 8% (wt/vol) polyethylene glycol 8000 (PEG8000 in PBS buffer) overnight at 4°C. Next, the ZIKV particles were collected by centrifugation at 10,414 × *g* for 50 min at 4°C. After resuspension, the ZIKV particles were purified through a 24% sucrose cushion using a Ti41 rotor (Beckman) at 105,400 × *g* for 1.5 h at 4°C. The purified ZIKV particles precipitates were then resuspended in 0.5 mL of Opti-MEM medium. After purification, the ZIKV particles were incubated with 1 μM ReAsH/FlAsH for 1 h at 37°C. The double-labeling was achieved by the addition of the membrane dye DiD (2 nM, Invitrogen) and incubation for 1 h at 37°C after FlAsH labeling. The free dye was removed through a PD MiniTrap G-25 column (GE Healthcare). The labeled ZIKVs were kept at 4°C and used within 48 h. Before confocal imaging, the labeled ZIKV particles were filtered through a 0.22-μm filter (Millipore). The labeled ZIKV particles were adsorbed on a glass coverslip for 30 min at 4°C and then were observed under a fluorescence microscope. To track the infection process of ZIKV, the cells were incubated with the labeled ZIKV particles for 10 min at 4°C and then transferred to a live-cell chamber preheated at 37°C for real-time imaging.

### Fluorescence imaging and statistical analysis.

Fluorescence images were captured using the Cytation 3 imaging reader (BioTek) or the Nikon A1 confocal microscope. For confocal imaging, the fluorescent emission was collected by a 60×, 1.2 NA oil immersion objective. The fluorescence images and videos were analyzed using Imaris software. The plaque sizes were analyzed using ImageJ software. The Mander’s correlation coefficients were calculated using the Nikon NIS-Elements analysis software.

## References

[1] White MK, Wollebo HS, David Beckham J, Tyler KL, Khalili K. 2016. Zika virus: an emergent neuropathological agent. Ann Neurol 80:479–489. 10.1002/ana.24748. 27464346 PMC5086418

[2] Pielnaa P, Al-Saadawe M, Saro A, Dama MF, Zhou M, Huang Y, Huang J, Xia Z. 2020. Zika virus: spread, epidemiology, genome, transmission cycle, clinical manifestation, associated challenges, vaccine and antiviral drug development. Virology 543:34–42. 10.1016/j.virol.2020.01.015. 32056845

[3] Sultan N, Bukhari SA, Ali I, Asif M, Umar Z, Akash MSH. 2018. Zika virus: a critical analysis and pharmaceutical perspectives. Crit Rev Eukaryot Gene Expr 28:357–371. 10.1615/CritRevEukaryotGeneExpr.2018025061. 30311585

[4] Ma Y, He Z, Tan T, Li W, Zhang Z, Song S, Zhang X, Hu Q, Zhou P, Wu Y, Zhang XE, Cui Z. 2016. Real-time imaging of single HIV-1 disassembly with multicolor viral particles. ACS Nano 10:6273–6282. 10.1021/acsnano.6b02462. 27253587

[5] Li Q, Li W, Yin W, Guo J, Zhang ZP, Zeng D, Zhang X, Wu Y, Zhang XE, Cui Z. 2017. Single-particle tracking of human immunodeficiency virus type 1 productive entry into human primary macrophages. ACS Nano 11:3890–3903. 10.1021/acsnano.7b00275. 28371581

[6] Yin W, Li W, Li Q, Liu Y, Liu J, Ren M, Ma Y, Zhang Z, Zhang X, Wu Y, Jiang S, Zhang XE, Cui Z. 2020. Real-time imaging of individual virion-triggered cortical actin dynamics for human immunodeficiency virus entry into resting CD4 T cells. Nanoscale 12:115–129. 10.1039/c9nr07359k. 31773115

[7] Li X, Wang D, Cui Z, Li Q, Li M, Ma Y, Hu Q, Zhou Y, Zhang XE. 2021. HIV-1 viral cores enter the nucleus collectively through the nuclear endocytosis-like pathway. Sci China Life Sci 64:66–76. 10.1007/s11427-020-1716-x. 32430850

[8] Li W, Liu J, Liu Y, Li Q, Yin W, Wanderi KK, Zhang X, Zhang Z, Zhang XE, Cui Z. 2021. HIV-1 uses dynamic podosomes for entry into macrophages. J Virol 95:e02480-20. 10.1128/JVI.02480-20. PMC8139672 33627394

[9] Li Y, Lu X, Li J, Bérubé N, Giest KL, Liu Q, Anderson DH, Zhou Y. 2010. Genetically engineered, biarsenically labeled influenza virus allows visualization of viral NS1 protein in living cells. J Virol 84:7204–7213. 10.1128/JVI.00203-10. 20463066 PMC2898259

[10] Hasan SS, Sevvana M, Kuhn RJ, Rossmann MG. 2018. Structural biology of Zika virus and other flaviviruses. Nat Struct Mol Biol 25:13–20. 10.1038/s41594-017-0010-8. 29323278

[11] Volkova E, Tsetsarkin KA, Sippert E, Assis F, Liu G, Rios M, Pletnev AG. 2020. Novel approach for insertion of heterologous sequences into full-length ZIKV genome results in superior level of gene expression and insert stability. Viruses 12:61. 10.3390/v12010061. PMC7019263 31947825

[12] Baker C, Xie X, Zou J, Muruato A, Fink K, Shi PY. 2020. Using recombination-dependent lethal mutations to stabilize reporter flaviviruses for rapid serodiagnosis and drug discovery. EBioMedicine 57:102838. 10.1016/j.ebiom.2020.102838. 32574959 PMC7317239

[13] Gadea G, Bos S, Krejbich-Trotot P, Clain E, Viranaicken W, El-Kalamouni C, Mavingui P, Desprès P. 2016. A robust method for the rapid generation of recombinant Zika virus expressing the GFP reporter gene. Virology 497:157–162. 10.1016/j.virol.2016.07.015. 27471954

[14] Shan C, Xie X, Muruato AE, Rossi SL, Roundy CM, Azar SR, Yang Y, Tesh RB, Bourne N, Barrett AD, Vasilakis N, Weaver SC, Shi PY. 2016. An infectious cDNA clone of Zika virus to study viral virulence, mosquito transmission, and antiviral inhibitors. Cell Host Microbe 19:891–900. 10.1016/j.chom.2016.05.004. 27198478 PMC5206987

[15] Adams SR, Tsien RY. 2008. Preparation of the membrane-permeant biarsenicals FlAsH-EDT2 and ReAsH-EDT2 for fluorescent labeling of tetracysteine-tagged proteins. Nat Protoc 3:1527–1534. 10.1038/nprot.2008.144. 18772880 PMC2843588

[16] Liu SL, Wang ZG, Xie HY, Liu AA, Lamb DC, Pang DW. 2020. Single-virus tracking: from imaging methodologies to virological applications. Chem Rev 120:1936–1979. 10.1021/acs.chemrev.9b00692. 31951121 PMC7075663

[17] Rudner L, Nydegger S, Coren LV, Nagashima K, Thali M, Ott DE. 2005. Dynamic fluorescent imaging of human immunodeficiency virus type 1 gag in live cells by biarsenical labeling. J Virol 79:4055–4065. 10.1128/JVI.79.7.4055-4065.2005. 15767407 PMC1061570

[18] Bussiere LD, Choudhury P, Bellaire B, Miller CL. 2017. Characterization of a replicating mammalian orthoreovirus with tetracysteine-tagged μNS for live-cell visualization of viral factories. J Virol 91:e01371-17. 10.1128/JVI.01371-17. 28878073 PMC5660500

[19] Tan TY, Fibriansah G, Kostyuchenko VA, Ng TS, Lim XX, Zhang S, Lim XN, Wang J, Shi J, Morais MC, Corti D, Lok SM. 2020. Capsid protein structure in Zika virus reveals the flavivirus assembly process. Nat Commun 11:895. 10.1038/s41467-020-14647-9. 32060358 PMC7021721

[20] Sotcheff S, Routh A. 2020. Understanding flavivirus capsid protein functions: the tip of the iceberg. Pathogens 9:42. 10.3390/pathogens9010042. PMC7168633 31948047

[21] Martin BR, Giepmans BN, Adams SR, Tsien RY. 2005. Mammalian cell-based optimization of the biarsenical-binding tetracysteine motif for improved fluorescence and affinity. Nat Biotechnol 23:1308–1314. 10.1038/nbt1136. 16155565

[22] Chang K, Baginski J, Hassan SF, Volin M, Shukla D, Tiwari V. 2016. Filopodia and viruses: an analysis of membrane processes in entry mechanisms. Front Microbiol 7:300. 27014223 10.3389/fmicb.2016.00300 PMC4785137

[23] Sakin V, Paci G, Lemke EA, Müller B. 2016. Labeling of virus components for advanced, quantitative imaging analyses. FEBS Lett 590:1896–1914. 10.1002/1873-3468.12131. 26987299

[24] Pomorski A, Krężel A. 2020. Biarsenical fluorescent probes for multifunctional site-specific modification of proteins applicable in life sciences: an overview and future outlook. Metallomics 12:1179–1207. 10.1039/d0mt00093k. 32658234

[25] Charlier CM, Wu YJ, Allart S, Malnou CE, Schwemmle M, Gonzalez-Dunia D. 2013. Analysis of borna disease virus trafficking in live infected cells by using a virus encoding a tetracysteine-tagged P protein. J Virol 87:12339–12348. 10.1128/JVI.01127-13. 24027309 PMC3807882

[26] Shi Y, Gao GF. 2017. Structural biology of the Zika virus. Trends Biochem Sci 42:443–456. 10.1016/j.tibs.2017.02.009. 28318966

[27] Li T, Zhao Q, Yang X, Chen C, Yang K, Wu C, Zhang T, Duan Y, Xue X, Mi K, Ji X, Wang Z, Yang H. 2018. Structural insight into the Zika virus capsid encapsulating the viral genome. Cell Res 28:497–499. 10.1038/s41422-018-0007-9. 29467384 PMC5939043

[28] Shang Z, Song H, Shi Y, Qi J, Gao GF. 2018. Crystal structure of the capsid protein from Zika virus. J Mol Biol 430:948–962. 10.1016/j.jmb.2018.02.006. 29454707

[29] Liu ZY, Li XF, Jiang T, Deng YQ, Zhao H, Wang HJ, Ye Q, Zhu SY, Qiu Y, Zhou X, Qin ED, Qin CF. 2013. Novel *cis*-acting element within the capsid-coding region enhances flavivirus viral-RNA replication by regulating genome cyclization. J Virol 87:6804–6818. 10.1128/JVI.00243-13. 23576500 PMC3676100

[30] Baker C, Liu Y, Zou J, Muruato A, Xie X, Shi PY. 2020. Identifying optimal capsid duplication length for the stability of reporter flaviviruses. Emerg Microbes Infect 9:2256–2265. 10.1080/22221751.2020.1829994. 32981479 PMC7594839

[31] Pierson TC, Diamond MS, Ahmed AA, Valentine LE, Davis CW, Samuel MA, Hanna SL, Puffer BA, Doms RW. 2005. An infectious West Nile virus that expresses a GFP reporter gene. Virology 334:28–40. 10.1016/j.virol.2005.01.021. 15749120

[32] Goh GK, Dunker AK, Uversky VN. 2016. Correlating flavivirus virulence and levels of intrinsic disorder in shell proteins: protective roles *vs*. immune evasion. Mol Biosyst 12:1881–1891. 10.1039/C6MB00228E. 27102744

[33] Yang MR, Lee SR, Oh W, Lee EW, Yeh JY, Nah JJ, Joo YS, Shin J, Lee HW, Pyo S, Song J. 2008. West Nile virus capsid protein induces p53-mediated apoptosis via the sequestration of HDM2 to the nucleolus. Cell Microbiol 10:165–176. 10.1111/j.1462-5822.2007.01027.x. 17697133 PMC7162166

[34] Slomnicki LP, Chung DH, Parker A, Hermann T, Boyd NL, Hetman M. 2017. Ribosomal stress and Tp53-mediated neuronal apoptosis in response to capsid protein of the Zika virus. Sci Rep 7:16652. 10.1038/s41598-017-16952-8. 29192272 PMC5709411

[35] Colpitts TM, Barthel S, Wang P, Fikrig E. 2011. Dengue virus capsid protein binds core histones and inhibits nucleosome formation in human liver cells. PLoS One 6:e24365. 10.1371/journal.pone.0024365. 21909430 PMC3164731

[36] Counihan NA, Rawlinson SM, Lindenbach BD. 2011. Trafficking of hepatitis C virus core protein during virus particle assembly. PLoS Pathog 7:e1002302. 10.1371/journal.ppat.1002302. 22028650 PMC3197604

[37] Delvecchio R, Higa L, Pezzuto P, Valadão A, Garcez P, Monteiro F, Loiola E, Dias A, Silva F, Aliota M, Caine E, Osorio J, Bellio M, O’Connor D, Rehen S, de Aguiar R, Savarino A, Campanati L, Tanuri A. 2016. Chloroquine, an endocytosis blocking agent, inhibits Zika virus infection in different cell models. Viruses 8:322. 10.3390/v8120322. PMC5192383 27916837

[38] Owczarek K, Chykunova Y, Jassoy C, Maksym B, Rajfur Z, Pyrc K. 2019. Zika virus: mapping and reprogramming the entry. Cell Commun Signal 17:41. 10.1186/s12964-019-0349-z. 31053158 PMC6500006

[39] Li M, Zhang D, Li C, Zheng Z, Fu M, Ni F, Liu Y, Du T, Wang H, Griffin GE, Zhang M, Hu Q. 2020. Characterization of Zika virus endocytic pathways in human glioblastoma cells. Front Microbiol 11:242. 10.3389/fmicb.2020.00242. 32210929 PMC7069030

[40] Brandenburg B, Zhuang X. 2007. Virus trafficking – learning from single-virus tracking. Nat Rev Microbiol 5:197–208. 10.1038/nrmicro1615. 17304249 PMC2740720

[41] Chiu CF, Chu LW, Liao IC, Simanjuntak Y, Lin YL, Juan CC, Ping YH. 2020. The mechanism of the Zika virus crossing the placental barrier and the blood-brain barrier. Front Microbiol 11:214. 10.3389/fmicb.2020.00214. 32153526 PMC7044130

[42] Mutso M, St. John JA, Ling ZL, Burt FJ, Poo YS, Liu X, Žusinaite E, Grau GE, Hueston L, Merits A, King NJC, Ekberg JAK, Mahalingam S. 2020. Basic insights into Zika virus infection of neuroglial and brain endothelial cells. J Gen Virol 101:622–634. 10.1099/jgv.0.001416. 32375993 PMC7414445

[43] Mladinich MC, Schwedes J, Mackow ER. 2017. Zika virus persistently infects and is basolaterally released from primary human brain microvascular endothelial cells. mBio 8:e00952-17. 10.1128/mBio.00952-17. 28698279 PMC5513708

[44] Oliveira ERA, Mohana-Borges R, de Alencastro RB, Horta BAC. 2017. The flavivirus capsid protein: structure, function and perspectives towards drug design. Virus Res 227:115–123. 10.1016/j.virusres.2016.10.005. 27751882

[45] Li Y, Li LF, Yu S, Wang X, Zhang L, Yu J, Xie L, Li W, Ali R, Qiu HJ. 2016. Applications of replicating-competent reporter-expressing viruses in diagnostic and molecular virology. Viruses 8:127. 10.3390/v8050127. PMC4885082 27164126

[46] Wen D, Li S, Dong F, Zhang Y, Lin Y, Wang J, Zou Z, Zheng A. 2018. *N*-Glycosylation of viral E protein is the determinant for vector midgut invasion by flaviviruses. mBio 9:e00046-18. 10.1128/mBio.00046-18. 29463651 PMC5821097

[47] Liu ZY, Yu JY, Huang XY, Fan H, Li XF, Deng YQ, Ji X, Cheng ML, Ye Q, Zhao H, Han JF, An XP, Jiang T, Zhang B, Tong YG, Qin CF. 2017. Characterization of *cis*-acting RNA elements of Zika virus by using a self-splicing ribozyme-dependent infectious clone. J Virol 91:e00484-17. 10.1128/JVI.00484-17. 28814522 PMC5640849

