# Supplementary material for: Tracking the Replication-Competent Zika Virus with Tetracysteine-Tagged Capsid Protein in Living Cells
Source: J Virol. 2022 Mar 14;96(7):e01846-21. doi: 10.1128/jvi.01846-21 (PMC9006885; doi:10.1128/jvi.01846-21)
Supplement: SUPPLEMENTAL FILE 1 — Supplemental Movie Legends. Download jvi.01846-21-s0001.pdf, PDF file, 0.09 MB [file jvi.01846-21-s0001.pdf]

SUPPLEMENTAL INFORMATIONS

Movie S1 Real-time imaging of the TC27 capsid protein in living cells. Vero cells were infected with the NR-TC27 ZIKV. The TC27 capsid proteins, pulse-chase labeled with ReAsH (red), were continuously tracked for 201 min. Nuclei were stained with Hoechst 33258 (blue).

Movie S2 Real-time imaging of the distribution of the TC27 capsid proteins expressed at different time. The previously expressed TC27 capsid proteins were labeled with FlAsH (green), and the newly expressed TC27 capsid proteins were pulse-chase labeled with ReAsH (red) in the infected cells.

Movie S3 Real-time imaging of single ReAsH-labeled NR-TC27 ZIKV particle moving along cellular filopodia. hCMEC/D3$^{Lifeact\text{-}EGFP}$ cells were infected with the ReAsH-labeled NR-TC27 ZIKV (red), and the process of single NR-TC27 ZIKV particle moving along cellular filopodia was imaged in real time.

Movie S4 Real-time imaging of single double-labeled NR-TC27 ZIKV particle entering a hCMEC/D3 cell. The single NR-TC27 ZIKV particle was labeled with FlAsH and DiD. Red: DiD labeled envelope; Green: FlAsH labeled capsid.

Movie S5 Real-time imaging of the infection process of single double-labeled NR-TC27 ZIKV. The single NR-TC27 ZIKV particle was labeled with FlAsH and DiD. Red: DiD labeled envelope; Green: FlAsH labeled capsid.
